# The Association Between Depression and Headache Characteristics in Adults

**DOI:** 10.1192/j.eurpsy.2026.10619

**Published:** 2026-07-31

**Authors:** Z. Aliyev, S. Aliyeva

**Affiliations:** 1 Psychiatry; 2 Neurology, Azerbaijan Medical University, Baku, Azerbaijan

## Abstract

**Introduction:**

Depression and headaches are among the most common global health burdens, often co-occurring and significantly impairing individuals’ quality of life. Emerging evidence suggests a bidirectional relationship between mood disorders and chronic pain syndromes, particularly tension-type headaches (TTH) and migraines. Understanding the interrelationship between depression and headache characteristics may inform more effective, integrated treatment approaches.

**Objectives:**

To investigate the association between depression and headache frequency, intensity, and type in adult populations, and to explore shared pathophysiological mechanisms contributing to comorbidity.

**Methods:**

A cross-sectional observational study was conducted with 250 adults aged 18–65, recruited from primary care and neurology clinics. Participants completed the Beck Depression Inventory-II (BDI-II) and the Headache Impact Test-6 (HIT-6). Depression diagnoses were confirmed using DSM-5 criteria, and headache types were classified per the International Classification of Headache Disorders (ICHD-3). Statistical analysis included Pearson correlation and multiple regression using SPSS version 26.0.

**Results:**

Depression severity was significantly positively correlated with both headache intensity and frequency (r = 0.62, p < 0.01). Participants with moderate to severe depression showed a higher prevalence of migraines and experienced diminished response to conventional headache treatments. Comorbid individuals reported higher HIT-6 scores, reflecting greater headache-related disability.

**Conclusions:**

The findings highlight a strong association between depression and chronic headache parameters, suggesting overlapping neurobiological mechanisms, such as serotonin dysregulation and HPA axis dysfunction. Routine screening for depressive symptoms in headache patients—and vice versa—is recommended to improve diagnosis and treatment outcomes. Integrated, multidisciplinary management strategies may yield better clinical results. Further longitudinal research is warranted to examine causality and therapeutic interventions targeting both disorders.

**Disclosure of Interest:**

None Declared

